# Topological Encoded Vector Beams for Monitoring Amyloid‐Lipid Interactions in Microcavity

**DOI:** 10.1002/advs.202100096

**Published:** 2021-05-02

**Authors:** Chaoyang Gong, Zhen Qiao, Zhiyi Yuan, Shih‐Hsiu Huang, Wenjie Wang, Pin Chieh Wu, Yu‐Cheng Chen

**Affiliations:** ^1^ School of Electrical and Electronic Engineering Nanyang Technological University 50 Nanyang Avenue Singapore 639798 Singapore; ^2^ Department of Photonics National Cheng Kung University Tainan 70101 Taiwan; ^3^ Key Lab of Advanced Transducers and Intelligent Control System of Ministry of Education Taiyuan University of Technology Taiyuan 030024 P. R. China; ^4^ School of Chemical and Biomedical Engineering Nanyang Technological University 62 Nanyang Drive Singapore 637459 Singapore

**Keywords:** amyloid‐lipid interaction, laser modes, liquid crystals, microcavity, topological structures, vector beams

## Abstract

Lasers are the pillars of modern photonics and sensing. Recent advances in microlasers have demonstrated its extraordinary lasing characteristics suitable for biosensing. However, most lasers utilized lasing spectrum as a detection signal, which can hardly detect or characterize nanoscale structural changes in microcavity. Here the concept of amplified structured light‐molecule interactions is introduced to monitor tiny bio‐structural changes in a microcavity. Biomimetic liquid crystal droplets with self‐assembled lipid monolayers are sandwiched in a Fabry–Pérot cavity, where subtle protein‐lipid membrane interactions trigger the topological transformation of output vector beams. By exploiting Amyloid *β* (A*β*)‐lipid membrane interactions as a proof‐of‐concept, it is demonstrated that vector laser beams can be viewed as a topology of complex laser modes and polarization states. The concept of topological‐encoded laser barcodes is therefore developed to reveal dynamic changes of laser modes and A*β*‐lipid interactions with different A*β* assembly structures. The findings demonstrate that the topology of vector beams represents significant features of intracavity nano‐structural dynamics resulted from structured light‐molecule interactions.

## Graphical abstract

The potential of vector laser beams is demonstrated to detect tiny bio‐structural changes in a microcavity through structured light‐molecule interactions. The topology of laser modes is converted into laser barcodes to reveal dynamic changes of amyloid‐lipid membrane interactions. Kinetic fingerprints of laser barcodes thus offer the potential for studying biophysical interactions in a wide range of biomedical applications.

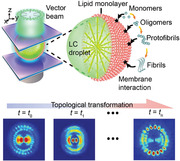

## 1 Introduction

Optical microcavities have emerged as a powerful tool over the past decade, attracting widespread attention for their prospects in biomedical and biological applications.^[^ ^1^, ^2^, ^3^, ^4^, ^5^, ^6^ ^]^ Thanks to the strong light‐molecule interaction induced by microcavity, subtle changes within the cavity could be detected.^[^ ^3^, ^4^, ^5^, ^6^ ^]^ Recently, microlasers from bio‐integrated systems have demonstrated distinct advantages for biochemical analysis in terms of signal amplification, narrow linewidth, and strong intensity, leading to higher sensitivity.^[^ ^6^, ^7^, ^8^ ^]^ Since the first demonstration of bio‐integrated laser,^[^ ^9^ ^]^ lasing has been reported in tissues,^[^ ^10^, ^11^ ^]^ cells,^[^ ^12^, ^13^, ^14^, ^15^, ^16^, ^17^, ^18^, ^19^ ^]^ biomolecules,^[^ ^20^, ^21^, ^22^, ^23^, ^24^, ^25^, ^26^, ^27^ ^]^ and living animals.^[^ ^28^ ^]^ Most studies utilized the lasing spectra as a sensing signal, where lasing frequency and intensity could provide information of averaged refractive index and optical loss in a cavity. However, the ability to detect tiny structural changes in microcavity is very limited. In contrast to lasing spectra (longitudinal mode), spatial images derived from transverse laser mode could provide information on the spatial distribution of physical properties and structural changes in microcavities.

Structured light plays a critical role in modern photonics for its flexibility in spatial intensity, phase, and polarization distribution.^[^ ^29^, ^30^, ^31^, ^32^ ^]^ In particular, vector beams with spatially dependent polarization have been extensively explored in areas such as laser micromachine,^[^ ^33^, ^34^ ^]^ particle manipulating,^[^ ^35^, ^36^ ^]^ high‐security encryption,^[^ ^37^, ^38^ ^]^ and super‐resolution imaging.^[^ ^39^, ^40^ ^]^ One of the most significant features of structured light is the high dependence of the topology on the structural changes, which offers a potential to expand the application of structured light for sensing.^[^ ^29^, ^41^, ^42^ ^]^ Generating structured light within laser cavity therefore enables stronger light‐matter interaction, which is extremely sensitive to the dynamic changes of physical structures encapsulated in the cavity.^[^ ^29^ ^]^

**Figure 1:**
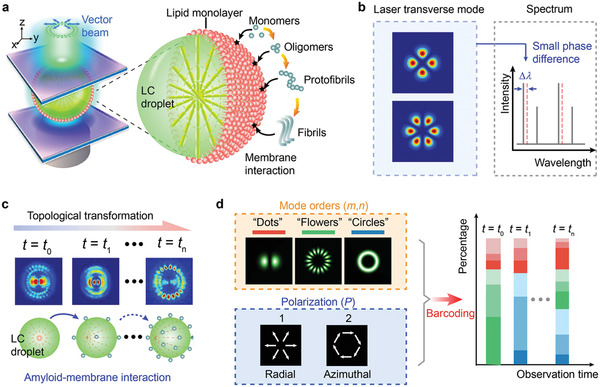
Concept of topological‐encoded laser barcode driven by molecular interactions. a) Schematic illustration of generating a vector beam driven by molecular interaction. Inset, illustration of mechanism. A*β* with different assemblies (monomers, oligomers, protofibrils, and fibrils) interact with the lipid monolayer coated on the LC droplet to trigger the topological transformation of the vector beam. b) Comparison of laser mode with conventional spectra interrogation. A small variation in intracavity phase can be detected by the topological transformation of transverse mode, however, the induced slight wavelength shift (Δ*λ*) can hardly be recognized in spectrum. c) Schematic illustration of topological transformation in laser mode pattern. The molecular interaction on the LC droplet surface was amplified by the LC molecules and result in the topological transformation of laser mode. d) Illustration of the developed encoding rule. The observed laser mode pattern can be decomposed into Laguerre–Gaussian (LG) mode with different orders and polarization states, which was further encoded into the barcode.

Herein, we explored the potential of using vector beams to monitor dynamic molecular interactions by tracing the topological features of output vector beam (laser mode order and polarization) generated from the liquid crystal (LC) microlaser. **Figure** **1a** shows the schematic of the vector beam generator, in which a LC droplet was sandwiched in a Fabry–Pérot (FP) microcavity. Vector beams could be modulated through strong light‐molecular interactions in a microcavity. Compared to spectrum interrogation, the superiority of employing transverse laser modes to detect molecular interaction mainly lies in the ultrahigh sensitivity to the intracavity structural changes (Figure 1b). For example, the spatial distribution of two Laguerre–Gaussian (LG) modes with similar orders are highly overlapped; hence they experience a similar intracavity phase change. The small phase difference between the two LG modes results in a small wavelength shift (Δ*λ*), which is hard to distinguish among lasing spectra. Nonetheless, the topology transformation of laser modes can be easily recognized through imaging.

For conceptual demonstration, a self‐assembled protein Amyloid beta (A*β*) was employed due to its significant role in Alzheimer's disease and cell signaling. A monolayer lipid membrane coated on the LC droplet was used for emulating the biophysical properties of a cellular outlet membrane. Subtle structural changes on lipid membranes caused by amyloid‐lipid membrane interactions will be amplified by LC and transferred into different topologies of light polarization states and mode orders, resulting in a significant change in laser beam output (Figure 1c). To better visualize the dynamic changes of vector laser beams, we also proposed a laser mode encoding method. As shown in Figure 1d, the laser mode orders and polarization states of the components were transferred into the RGB barcode, which enabled better tracking of the temporal evolution of laser mode. Our findings reveal that different molecular concentrations and sizes would lead to different temporal switching of vector beam topologies in real‐time. The kinetic fingerprints of laser barcodes offer the potential for studying biophysical interactions in a wide range of biomedical applications.

## 2 Results

### 2.1 Laguerre–Gaussian Beams from Liquid Crystal Droplets

**Figure 2:**
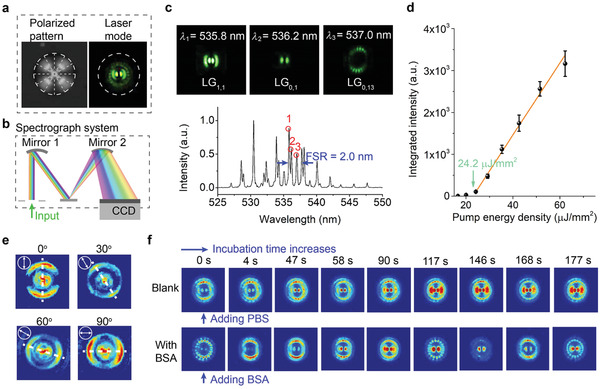
Observation of transverse laser mode from LC droplet. a) Image of LC droplets taken under polarized optical microscopy (left panel) and the corresponding transverse laser mode pattern (right panel). The dashed lines indicate the radial director configuration, and the dashed circles indicate the size of the LC droplet. b) Schematic illustration of the hyperspectral imaging system. The laser mode pattern was dispersed by a diffractive grating according to its spectral components. c) The hyperspectral images (top) extracted from the laser mode in (a) and the corresponding laser emission spectrum (bottom). The spectrum is recorded at a pump energy density of 51.5 µJ mm^−2^. d) The spectrally integrated intensity with various pump energy densities. The error bars are obtained based on three times measurements. e) Polarization‐dependent laser mode patterns after passing through a linear polarizer with different angles. The direction of the maximum intensity (dashed line) agrees well with the orientation of the linear polarizer (arrow). f) Comparison of the temporal evolution of laser mode without (top) or with (bottom) 15 × 10^–9^ m BSA.

We started by observing the laser mode from dye‐doped LC droplets in a FP cavity. The LC droplet with radial director configuration used in our experiment shows a typical polarized pattern (left, **Figure** **2a**). A distinctive pattern resulted from transverse mode can be observed when the LC droplet is excited in FP cavity (right, Figure 2a). The observed transverse mode is the solution of Maxwell's equation under the boundary condition imposed on the LC droplet, which is highly dependent on the intracavity structure of the droplet. As such, tiny structural changes on the LC droplet will lead to significant changes in laser mode pattern. To visualize each component of the laser mode pattern, we imaged the laser emission with a spectrograph system (Figure 2b and Figure S1, Supporting Information). The hyperspectral images at different wavelengths indicate that the laser mode pattern in Figure 2a can be viewed as the superposition of LG modes with various radial and azimuthal orders (top, Figure 2c). Meanwhile, the emission spectra show individual sharp peaks with a free spectral range of 2.0 nm (bottom, Figure 2c), corresponding to the longitudinal mode of laser emission. The lasing spectra and the corresponding integrated intensity under various pump energy densities are supported in Figure 2d and Figure S2, Supporting Information. Because of the lens effect provided by the LC droplet,^[^ ^43^ ^]^ a relatively low lasing threshold of 24.2 µJ mm^−2^ is obtained, which is comparable to the previous results.^[^ ^44^ ^]^

Laser vector beam emissions from the LC droplet were confirmed by tuning the orientation of the linear polarizer. As shown in Figure 2e, a spatial‐dependent polarization vector of the laser mode was attested. As such, the polarization vector offers one more degree of freedom to quantify the topological changes besides the order of laser modes. The spatial‐dependent polarization of laser emission is due to the 3D radial distributed optical axis in LC droplet (left, Figure 2a). The intracavity radial birefringence distribution determines the laser mode to be either radial or azimuthal polarized. Theoretical simulations of the vector beam from LC droplet could help resolve the complex 3D anisotropic structure.^[^ ^45^ ^]^ However, it is beyond the scope of our discussion.

To explore the topological changes of laser modes, protein molecules‐ bovine serum albumin (BSA)‐were employed to induce molecular interaction at the LC‐membrane interface. BSA can bind to the surfactant molecules on the LC droplet through electrostatic and hydrophobic interaction.^[^ ^46^, ^47^ ^]^ In the absence of BSA, the observed laser mode pattern is relatively stable, with only intensity variation induced by mode competition (top, Figure 2f). Upon binding to BSA, continuous topological transformation of laser modes was triggered (bottom, Figure 2f). The significant topological transformation of laser modes can be attributed to the ultrahigh sensitivity of transverse laser mode to the intracavity structure. The binding of BSA on the droplet surface‐induced slight disturbance in the orientation of LC molecules. Because of the strong birefringence of LC molecules, such disturbance could trigger the redistribution of refractive index in a microcavity, leading to different eigenmodes and enhanced the mode competition. Additionally, we investigated the impact of droplet sizes and cavity length on laser modes in Figures S3 and S4, Supporting Information. Detailed discussions on the influence of these two parameters were also provided.

In order to demonstrate the high sensitivity of laser mode pattern, we compared the laser mode evolution with the images obtained with polarized optical microscopy, which is the standard method for visualizing conformation changes of LC.^[^ ^48^, ^49^ ^]^ As illustrated in Figure S5, Supporting Information, after BSA (15 × 10^–9^ m) was induced, the polarized images remain stable during our measurement. Even at ten times higher BSA concentration (150 × 10^–9^ m), no significant change was observed until *t* = 183 s (Figure S6, Supporting Information). In contrast, the laser mode started to change at *t* = 9 s after BSA was induced. A faster transition between different patterns was also observed. The results indicate that laser mode possesses much higher sensitivity than conventional polarized images, which carries information about the dynamic molecular binding.

### 2.2 Investigation of Amyloid Peptide‐Lipid Membrane Interactions

**Figure 3:**
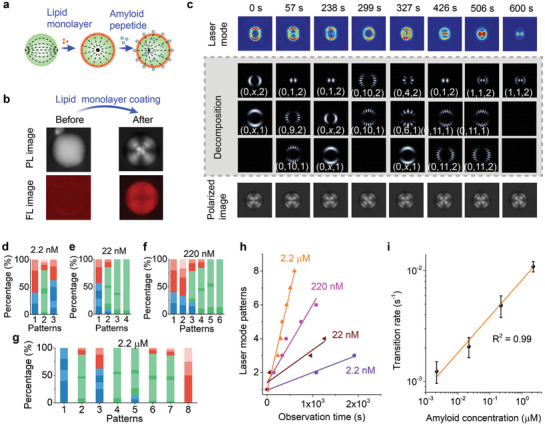
Topological transformation driven by Amyloid *β*‐lipid membrane interaction. a) Illustration of lipid monolayer coating and amyloid‐*β* (A*β*)‐lipid membrane interaction. b) Comparison of its corresponding polarized (PL) and fluorescence (FL) image before (left) and after (right) lipid monolayer coating. c) Comparison of the laser mode evolution and the polarized image. The laser mode decomposition results are also provided. The concentration of A*β* is 2.2 × 10^–6^ m. Topological laser mode barcode with A*β* concentration of d) 2.2 × 10^–9^ m, e) 22 × 10^–9^ m, f) 220 × 10^–9^ m, and g) 2.2 × 10^–6^ m, respectively. h) Comparison of laser mode temporal evolution under various A*β* concentrations. i) The transition rate as a function of A*β* concentrations.

Interaction between amyloids and cellular membrane plays a crucial role in Alzheimer's disease by lipid peroxidation and forming ion‐permeable pores on the cellular membrane.^[^ ^50^, ^51^, ^52^, ^53^ ^]^ However, conventional approaches are generally limited by their weak light‐matter interactions to capture the nanoscale structural changes of the cellular membrane at an early stage. In this section, we show how tiny structural changes on the lipid membrane influence the output vector beams. Herein, bionic cell model was built by coating a lipid monolayer on the LC droplet (**Figure** **3a**) to monitor the A‐*β*lipid membrane interactions. The lipid monolayer was used for emulating biophysical properties of a cellular outlet membrane. The peptide‐lipid membrane interaction triggers the molecular orientation of LC, and eventually, the interaction signals were converted to the topological transformation of laser mode. The coating of lipid monolayer was confirmed by the bipolar to radial conformation changes and the presence of strong fluorescence emission (Figure 3b). Proteins like BSA can interact with the lipid molecules through electrostatic attraction. The interactions between a lipid membrane and BSA are determined by its physico‐chemical characteristics, mainly their dipolar character (or charged patches).^[^ ^54^ ^]^ Compared to uncoated LC droplets, the lipid monolayer‐coated LC droplet showed a higher sensitivity to molecular binding events (Figure S7, Supporting Information), which is likely the result of stronger molecular interactions between lipid and BSA.^[^ ^54^, ^55^ ^]^ This agrees with previous literature where LCs could amplify conformational changes resulted from electrostatic binding.^[^ ^24^, ^56^ ^]^

Once amyloid peptide was induced, a continuous topological transformation in laser modes can be observed (top, Figure 3c) due to tiny membrane structural changes. Strong evidence in the literature has shown that the amyloid‐lipid interaction is strongly related to the electrostatic and hydrophobic interaction,^[^ ^52^, ^57^ ^]^ in which the electrostatic interaction between charged peptide residues and lipid headgroups modulate the further penetration of the C‐termini of amyloids into the hydrophobic region of lipid membranes. The topology of laser mode patterns can then be decomposed into three topological components, including “dot,” “flower,” and “circles.” For each topological component, the laser mode orders and polarization state can be recognized (Figures S8–S10, Supporting Information). We defined (*m*, *n*, *P*) to characterize the significant divergence of laser mode orders and polarization state (bottom, Figure 3c), where *m*, *n* denotes the radial and azimuthal indices of laser mode. *P* = 1, 2 corresponds to azimuthal and radial polarization, respectively. The results indicate that the observed laser mode patterns appear to be a random combination of LG modes with different orders and polarization. Such dynamic changes of topological laser mode can be attributed to the competition between different eigenmode families.

Subsequently, we investigated the topological evolution of vector beams under various A*β* concentrations and converted them into representative topological laser barcodes. Detailed decomposition results of vector beams are provided in Tables S1–S4 and Figures S11–S13, Supporting Information, where the laser mode orders and polarization were recorded under various A*β* concentrations. The laser mode patterns were converted to barcode by calculating the percentage of a specific element in the decomposition results using $\eta_{i,j}=N_{i,j}/\sum_{i=1}^{K}\sum_{j=1}^{3}N_{i,j}\times \;100\%$, with *i* = 1, 2,…, *K* and *j* = 1, 2, 3. Here, *N*_*i*,1_ = *m_i_*, *N*_*i*,2_ = *n_i_*, *N*_*i*,3_ = *P_i_* denotes the laser mode orders and polarization state of the *i* th decomposition. *K* means the number of the mode decomposition (see details in Experimental Section). We use RGB colors to represent different types of topological components and different color depths were employed to characterize the significant divergence of laser mode orders and polarization states. The resulting RGB barcodes are shown in Figure 3d–g. The information that can be derived from the barcodes includes three aspects. First, the type of topological components in each laser mode pattern can be intuitively revealed by RGB color, where red (R) denotes “dot,” green (G) denotes “flower” and blue (B) denotes “circles,” respectively. Second, the evolution of each topological component in orders and polarization state can be obtained by comparing the barcode with the adjacent ones. Third, the number of observed laser mode patterns with various A*β* concentrations can be obtained by counting the barcodes over time. The obtained barcode, therefore, serves as a “fingerprint” of the laser mode, which clearly shows the topological information of laser modes and enables tracing of the dynamic peptide‐lipid interactions.

The challenges of employing the laser mode pattern to monitor molecular interactions lie in two aspects. First, our results show that laser modes are extremely sensitive to the geometrical structures, which can be a disadvantage (repeatability) or advantage (high sensitivity). Even two nearly identical droplets can provide significantly different lasing modes under slight disturbances. Second, mode competition occurs between modes sharing similar transverse intensity profiles, hence results in different temporal evolution between similar droplets. Fortunately, by recording the onset time of each topological laser mode (the time when the laser mode starts to change), we observed good linearity between the number of observed laser mode patterns and the onset time (Figure 3h). A more frequent transition in laser mode is observed when a higher A*β* concentration was induced, indicating a stronger A*β*‐lipid membrane interaction. By defining the transition rate (i.e., the number of observed laser mode patterns per second), the response of transverse lasers mode to different A*β* concentrations are calibrated in Figure 3i, showing good linearity (*R* = 0.99) and reproducibility. These findings confirm that the laser mode possesses the capability to identify the strength of molecular interaction through time. As shown in Figure 3i, the limit of detection (LOD) for A*β* was calculated to be 2.5 × 10^–9^ m by using LOD = 3*α*/*k*, where *α* is the standard deviation, and *k* is the slope. Note that such performance is comparable with the A*β* level in cerebrospinal fluid in Alzheimer's disease patients.^[^ ^58^ ^]^

### 2.3 Laser Mode Monitoring of Amyloid Fibrillization

**Figure 4:**
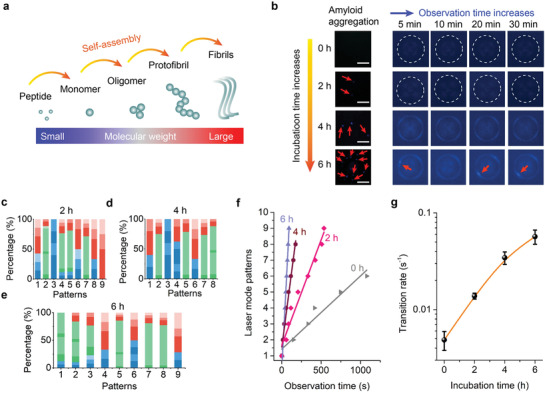
Laser mode analysis of different amyloid‐*β* assembly structures interacting with lipid membrane. a) Illustration of A*β* self‐assembly structures. b) Left panel: Fluorescence microscopic images of A*β*‐lipid membrane interaction under different incubation times (0, 2, 4, 6 h) and observation times (5, 10, 20, 30 min). Scale bars, 10 µm. Topological laser barcodes converted from A*β*‐lipid membrane interaction under different incubation times of c) 2 h, d) 4 h, e) 6 h. f) Comparison of laser mode temporal evolution with different A*β* assembly structures interacting with lipid membranes. g) The transition rate as a function as incubation time.

Last, we investigated how interactions between different A*β* assembly structures and lipid membranes may change the output vector beams. Literature has shown that the self‐assembly of amyloid peptide into higher‐order structures correlates with higher neurotoxicity in Alzheimer's disease pathology.^[^ ^50^, ^59^ ^]^ As such, investigating the response of laser mode to different A*β* assembly structures may provide a new approach to reveal the pathogenesis of Alzheimer's diseases at the molecular level. The illustration in **Figure** **4a** shows A*β* assembly structures under different stages, corresponding to different neuron toxicity and responses. Different A*β* assemblies were obtained by incubating the A*β* peptide solution at 37 °C with different reaction times (see Experimental Section). Figure 4b shows the aggregation of A*β* under different reaction times by imaging with fluorescence microscopy. The insoluble fibril emerged on the LC droplet surface with increased fluorescence after 4 h of assembly. Enlarged fibrils were observed after 6 h of assembly, forming bright clusters on the droplet surface. The location of the bright spot slightly changes over time owing to the liquidity of the LC and the strong molecular interaction between A*β* and lipid membrane.

Dynamic laser modes were monitored through incubation time under a fixed A*β* concentration. Contrary to the fluorescence microscopic images in Figure 4b, the laser mode shows much higher sensitivity and signal to noise ratio (Figures S14–S16, Supporting Information). The corresponding barcodes with various A*β* assembly structures are plotted in Figure 4c–e, through which we can derive the details of the dynamic evolution of the topological laser mode. Figure 4f depicts the laser modes as a function of time with different A*β* assembly structures, where the number of observed laser mode patterns and the transition rate increased through time. The transition rates with various incubation times extracted from Figure 4f are shown in Figure 4g. The deviation of linearity may be attributed to the increased amount of insoluble higher order A*β* aggregates after 6 h of reaction, which has been reported to be less neurotoxic.^[^ ^59^ ^]^

## 3 Discussion

Since the first invention of laser emission‐based imaging a few years ago, most studies focused on the generation of laser emission as a detection signal or enhanced resolution approach. Many types of optical microcavities, including FP cavity, whispering‐gallery mode microcavity, plasmonics, and photonic crystals, have been widely employed for lasing and bioassay.^[^ ^18^, ^60^, ^61^, ^62^, ^63^ ^]^ In particular, FP cavity provides a whole‐body interaction between the light and the gain medium, in contrast to the evanescent interaction in ring resonator sensors or gold plasmonic sensors. Limited by the resonance mechanism, observing transverse mode in plasmonics and ring resonators is nearly impossible. With the advantage of the planar configuration, FP cavity is particularly attractive for laser mode imaging. To the best of our knowledge, this study represents the first study to image structured light‐molecule interactions through laser modes. This study shows that vector beams can be viewed as a topology of laser modes composed of different mode orders and polarization states. The unique topological laser barcodes derived from laser modes could serve as a fingerprint to provide a more intuitive way for tracing the topological evolution. Our results demonstrated that vector beams show a high sensitivity to biomolecules, which can potentially reveal the physiological mechanism of A*β*‐lipid membrane interaction at an early stage. The concentration, molecular size, and assembly structures of A*β* were also found to influence the molecular interaction between A*β* and lipid membrane.

To date, numerous technologies have been developed for identifying molecular signatures on the membrane of live cells, such as Mechanical Trap Surface‐Enhanced Raman Spectroscopy and atomic force microscopy.^[^ ^64^, ^65^, ^66^, ^67^, ^68^ ^]^ Such methods provide a powerful tool to obtain 3D profiling of biochemical contents (amino acids, lipids) localized on biological membrane layers. In addition to biochemical signals, our system is extremely sensitive to biophysical and structural changes, which may provide complementary information to biochemical signals.^[^ ^69^ ^]^ However, the shortage of our system is that it cannot directly interpret the chemical components of the surface interactions. Another challenge is that 3D imaging is not possible at this stage. Since the laser light inside a cavity is bounced back and forth between two mirrors, the laser output represents an accumulative effect in the pump direction. By using a concave mirror or 3D‐confined cube‐like cavity to recover the 3D information would be the next important step in the biolaser development. It should also be noted that an individual laser mode pattern in our configuration does not correspond to any state of the molecular interaction. This is because the laser mode pattern is significantly affected by the mode competition and the heterogeneity of the LC droplets. To increase the reproducibility of the data analysis, it is necessary to optimize the same lasing conditions. As laser is very sensitive to droplet structure, it is essential to make sure the LC droplets and cavity are identical as possible. To reduce variations, fabrication of the droplets can be solved by using microfluidics technique. The fixation of the laser cavity is also very critical; hence it may be possible to develop a fixed microfluidic cavity device when handling large amounts of biological droplets in the future.

In conclusion, we would like to provide several outlooks. First, this study suggests that transverse modes can carry spatial information of the intracavity refractive index profile, which is intrinsically different from the general information provided by the longitudinal mode. The concept of topological laser barcode further enhances the capability of using laser modes to study subtle changes within the microcavity. Although an artificial system was used in this system, the proposed approach can also be used to study live cell interactions. By chemically modifying the surface layer of LC droplets, LC droplets could be used to capture specific antigens released from live cells. LC droplets can also be designed to mimic different functions of a living cell according to different purposes. Second, the proposed approach can serve as a versatile platform for studying protein‐membrane interactions, particularly in neurodegenerative diseases and drug screening. The ability to enable label‐free detection provides the opportunity for a wide range of biological analyses. Third, this study offers new opportunities for multiplexed laser imaging. Taking advantage of the narrow linewidth of laser emissions, distinctive laser mode only emerges at specific lasing wavelengths. With the integration of the spectrograph system, laser mode images could also correspond to particular proteins by labeling with different fluorophores.

## 4 Experimental Section

##### Optical System Setup

The experimental setup is illustrated in Figure S1, Supporting Information. Two highly reflective customized mirrors with a reflectivity of 99.9% (500–600 nm) were used to form a FP cavity. The bottom mirror was treated with 0.01% (w/v) Poly‐L‐lysine for 20 min to immobilize the LC droplets through electrostatic attraction. The two mirrors were separated by glass beads with a diameter of 45 µm. A Nikon Ni‐E upright microscope with 10 × objective was used for excitation and signal collection.

For laser mode detection, a pulsed laser (EKSPLA PS8001DR, 50 Hz, 5 ns pulse width) integrated with an optical parametric oscillator (OPO) was used as an optical pump. The emission wavelength of OPO was fixed at 470 nm and focused on the LC, forming a beam size of a diameter of 31 µm. The laser emission was split by a beam splitter and sent to a spectrometer (Andor Kymera 328i) and charge‐coupled device (CCD‐1) (Andor Zyla SCMOS) for spectrum and image collection. The hyperspectral images were taken by choosing 1200 lines mm^−1^ grating and keeping the entrance slit of the spectrometer open. Because of the dispersion induced by the holographic grating, the spatial pattern associated with different wavelengths will be dispersed to the different locations of the CCD‐2. A band‐pass filter (BPF‐1) was used to filter out the influence of the pump laser. A linear polarizer (LP‐1) was used to check the polarization state of laser mode.

To obtain polarized images, the white light‐emitting diode (LED‐1) mounted on the microscope was employed as a light source. A linear polarizer (LP‐2) was inserted to generate a linear polarized light that was perpendicular to the LP‐1. All the polarized images were acquired by CCD‐1. For fluorescence imaging of lipid, the highly reflective mirrors were replaced with two pieces of glass slides. A broadband LED (SOLA) was used for excitation (LED‐2), and a filter (BPF‐2) was used to select the wavelength between 540 to 580 nm. For fluorescence observation A*β* aggregation, the excitation wavelength between 465 to 495 nm was selected. The fluorescence images were acquired using CCD‐1.

##### LC Droplets Generation

The nematic LC 4‐cyano‐4′‐pentylbiphenyl (Sigma, No. 328510) doped with 20 × 10^–3^ m fluorescent dye Coumarin 6 (Sigma, No. 442631) was used for droplet generation. The LC droplets were produced by mechanically mixing 10 µL dye‐doped LC with 1 mL sodium dodecyl sulfate (SDS) solution (Sigma, No. L3771). Droplets with a diameter of ≈25 µm were used throughout the experiment. Note that the concentration of the SDS solution differs in the experiments. For laser mode characterization and conceptual demonstration of laser mode encoding, 2 × 10^–3^ m SDS solution was used. For A*β*‐lipid membrane interaction detection, 200 × 10^–6^ m SDS was used.

##### Coating Lipid Monolayer on LC Droplets

The lipid 1‐palmitoyl‐2‐oleoyl‐glycerol‐3‐phosphocholine (POPC) and 1‐palmitoyl‐2‐oleoyl‐sn‐glycerol‐3‐phospho‐(1′‐rac‐glycerol) (POPG) was used in the experiment because they were representative of the biological membrane. The lipid powder of POPC (Avanti, No. 850457) and POPG (Avanti, No. 840457) was first dissolved in 2:1 (v/v) mixture of chloroform/methanol to a concentration of 2 × 10^–3^ m, respectively. Then, the POPC/POPG mixture was obtained by mixing POPC and POPG solution with 3:1 (mol/mol) and stored at −20 °C for future use.

For coating lipid monolayer on LC droplets, 80 µL POPC/POPG mixture was loaded in a 15 mL glass beaker and was immediately placed in a vacuum chamber for 4 h to remove all the solvents. The dried lipid film was subsequently suspended in 1 mL phosphate‐buffered saline (PBS, PH = 7.4). Liposome solution was produced by 30 min sonication at 45 °C. Lipid monolayer on LC droplets was self‐assembled by mixing liposome solution with LC droplets with 1:1 (v/v) and incubating for 5 min. The lipid‐coated LC droplets were freshly prepared in each measurement.

To image the monolayer lipid‐coated on LC droplets, lipophilic dye Nile Red (Sigma, No. N3013) with a concentration of 20 × 10^–6^ m was induced into the POPC/POPG mixture during liposome preparation. Furthermore, the nematic LC without Coumarin 6 was used for the LC droplets generation. The presence of the lipid on the LC droplet was confirmed by the presence of fluorescence emission on LC droplets.

##### Droplet Sorting and Transferring

The mechanical mixing method used for droplet generation was simple; however the size cannot be precisely controlled. In the experiment, no specific sorting method was employed. The LC droplets of different sizes were sandwiched in the FP cavity. LC droplets with different sizes were transferred onto the bottom mirror, where only droplets with 25 µm were selected diameter for laser mode observation. Then, the microbeads were added to the solution to form spacers. Subsequently, the top mirror was covered on top of the device. See Figure S1, Supporting Information.

##### Laser‐Mode Decomposition and Barcoding

The laser mode decomposition was conducted manually according to the spatial distribution of the laser pattern. Each laser pattern was decomposed into topological components, including “dots,” “flowers,” and “circles.” The laser mode orders and polarization states of the components were determined. Specifically, for the component “circles,” the azimuthal mode order cannot be precisely recognized, *x* was used instead. The laser mode patterns were subsequently transferred into RGB barcodes based on the decomposed results. The overall procedural consisted of 3 steps. Step 1: the temporal evolution of laser mode orders and polarization state was recorded in Tables S1–S7, Supporting Information. Step 2: the percentage of each cell in the table column was calculated by using $\eta_{i,j}=N_{i,j}/\sum_{i=1}^{K}\sum_{j=1}^{3}N_{i,j}\times 100\%$, with *i* = 1, 2,…, *K* and *j* = 1, 2, 3. Here, *N*_*i*,1_ = *m_i_*, *N*_*i*,2_ = *n_i_*, *N*_*i*,3_ = *P_i_* denotes the laser mode orders and polarization state of the *i* th decomposition. *K* denotes the number of the mode decomposition in the column. For simplification, *x*  =  1 was used in the calculation. Step 3: the result was transferred to the RGB barcodes where the length of each color denoted the percentage (*η*_*i*,*j*_) calculated in Step 2. The RGB color represented “dot,” “flower,” and “circles,” respectively. For each topological component, different color depths were employed to characterize the significant divergence of laser mode orders and polarization states.

##### Procedure for Amyloid Peptide Concentration and Different Assemblies

A*β* peptide stock solution was obtained by dissolving the lyophilized powder (Invitrogen, No. 03‐112) in dimethyl sulfoxide to a concentration of 2.2 × 10^–3^ m and stored at −20 °C. Standard A*β* peptide solution with concentration from 22 × 10^–9^ m to 22 × 10^–6^ m was obtained through tenfold serial dilution with PBS. The standard A*β* peptide solution was mixed with the lipid‐coated LC droplet with 1: 9 (v/v) and the final concentration of A*β* peptide was from 2.2 × 10^–9^ m to 2.2 × 10^–6^ m. The A*β* treated LC droplets were immediately sandwiched in the FP cavity for laser mode measurement. A*β* with various assemblies were obtained by incubating 22 × 10^–6^ m peptide with different times (0, 2, 4, 6 h) at 37 °C. Then, the A*β* was diluted ten times and ready for the measurement. The A*β* solution was subsequently mixed with the lipid‐coated LC droplet with 1: 9 (v/v) and the final concentration of A*β* was 220 × 10^–6^ m.

##### Fluorescence Observation of Amyloid Aggregation and Bindings

Thioflavin T (ThT) with a concentration of 1 × 10^–3^ m was freshly prepared by dissolving the powder (Tokyo Chemical Industry Co. Ltd., No. T0558) in DI water. Then, A*β* peptide with a concentration of 22 × 10^–6^ m was mixed with ThT solution with 9: 1 (v/v), followed by incubation at 37 °C for fibrillation. During the reaction, non‐fluorescent ThT specifically binds to the aggregated A*β* and shows strong fluorescence emission. After incubation, the mixture of A*β* and ThT was further diluted by tenfolds, and 10 µL mixture was used for fluorescence microscopy. In order to observe the fluorescence of A*β* on LC droplet, the LC without Coumarin 6 was used for droplet generation. After lipid coating, the droplets were ready to use. The mixture of A*β* and ThT was mixed with LC droplet at 1: 9 (v/v). The resulting final concentration of A*β* was 200 × 10^–9^ m. The final mixture was immediately sandwiched in two glass slides.

##### Statistical Analysis

All the laser threshold plots were based on an average of three measurements. For each experiment, at least three droplets were tested and compared in all datasets. All the laser mode transformations in Figures 2 and 3 were captured with monocolor CCD then transferred into heatmap color with MATLAB for enhanced visualization. The fluorescence images in Figure 4 were captured by monocolor CCD and filled in with pseudo color to represent the fluorescence signals.

## Conflict of Interest

The authors declare no conflict of interest.

## Author Contributions

Y.‐C.C. formulated the idea behind the study. C.G. and Y.‐C.C. designed experiments. C.G. conducted and performed the experiments. Z.Q. conducted the theoretical analysis for laser modes. Z.Y. performed LC droplets generation. S.‐H.H., W.W., and P.C.W. fabricated the mirror cavity. C.G. and Y.‐C.C. drafted the paper.

## Supporting information

Supporting Information

## Data Availability

The data that support the findings of this study are available from the corresponding author upon reasonable request.
