# Supplementary material for: Topological Encoded Vector Beams for Monitoring Amyloid‐Lipid Interactions in Microcavity
Source: Adv Sci (Weinh). 2021 May 2;8(12):2100096. doi: 10.1002/advs.202100096 (PMC8224421; doi:10.1002/advs.202100096)
Supplement: Supplementary file 1 — Supporting Information [file ADVS-8-2100096-s001.pdf]

© 2021 Wiley-VCH GmbH

ADVANCED SCIENCE Open Access

Supporting Information

for *Adv. Sci.,* DOI: 10.1002/advs.202100096

"Topological Encoded Vector Beams for Monitoring Amyloid-Lipid Interactions in Microcavity"

Chaoyang Gong, Zhen Qiao, Zhiyi Yuan, Shih-Hsiu Huang, Wenjie Wang, Pin Chieh Wu, Yu-Cheng Chen**

Supporting Information for

"Topological Encoded Vector Beams for Monitoring Amyloid-Lipid Interactions in Microcavity"

Chaoyang Gong[1], *Zhen Qiao*[1], *Zhiyi Yuan*[1], *Shih-Hsiu Huang*[2], *Wenjie Wang*[3], *Pin Chieh Wu*[2★], *Yu-Cheng Chen* [1,4★]

[1] School of Electrical and Electronic Engineering, Nanyang Technological University, 50 Nanyang Avenue, 639798, Singapore

[2] Department of Photonics, National Cheng Kung University, Tainan 70101, Taiwan

[3] Key Lab of Advanced Transducers and Intelligent Control System of Ministry of Education, Taiyuan University of Technology, 030024, P. R. China

[4] School of Chemical and Biomedical Engineering, Nanyang Technological University, 62 Nanyang Drive, 637459, Singapore

★Correspondence Email: pcwu@gs.ncku.edu.tw ; yucchen@ntu.edu.sg

CONTENTS

1 Characterization of laser emission
1.1 Experimental setup
1.2 Emission spectra with various pump energy densities
1.3 Influence of droplet size and cavity size on the laser mode pattern 4
1.4 Ultrahigh sensitivity of laser modes
2 Investigation of Amyloid peptide-lipid membrane interactions
2.1 Lipid coated LC droplets
2.2 Recognition of the laser mode topologies
2.3 Dynamic laser mode evolution with various Aβ peptide concentration
3 Laser mode monitoring of Amyloid fibrillization

1 CHARACTERIZATION OF LASER EMISSION

1.1 Experimental setup

The schematic illustration of the experimental setup was shown in Supplementary Fig. 1. Briefly, the laser emission from the LC droplet was split by a beam splitter and send to a CCD and a spectrometer, thus enabling the simultaneous collection of laser mode imaging and spectra.

The hyperspectral images were collected by keeping the slit widely open. The laser emission was diffracted using a grating and imaged by a thermoelectrically cooled CCD.

The resulting hyperspectral image shows separated laser mode components on a specific wavelength.

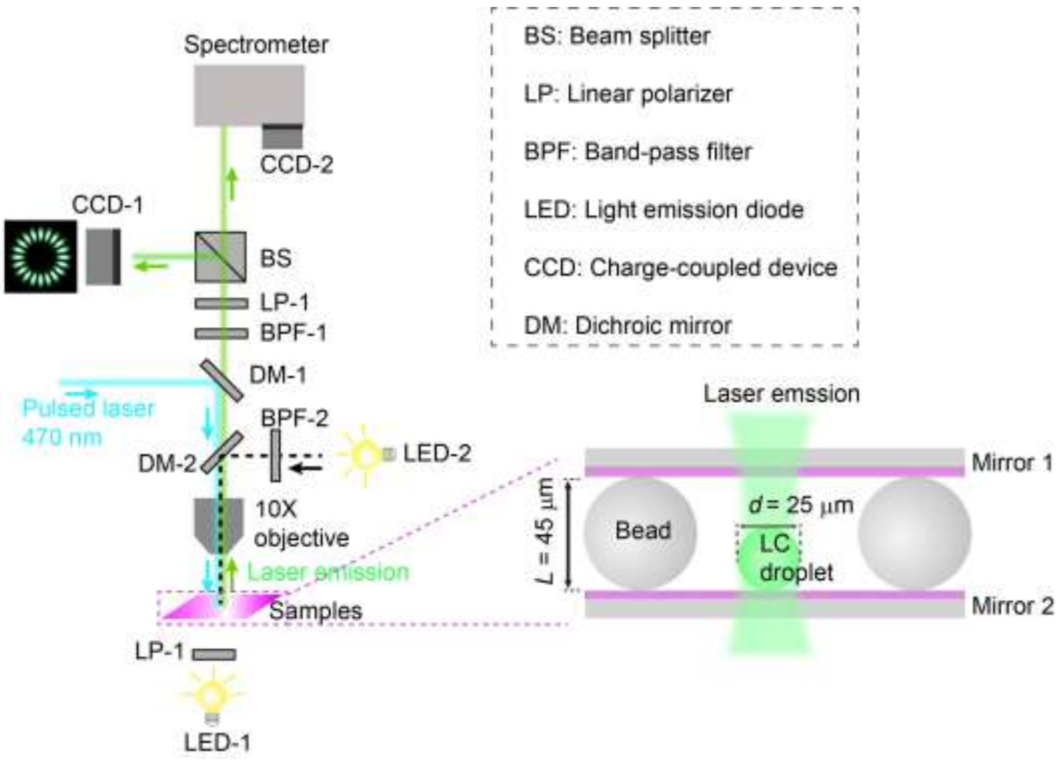

Supplementary Figure 1. Illustration of the experimental setup. Inset, the enlargement of the vector beam emitter.

1.2 Emission spectra with various pump energy densities

The emission spectra with various pump energy densities were plotted in Supplementary Fig. 2. The observed peaks in spectra correspond to the longitudinal laser modes.

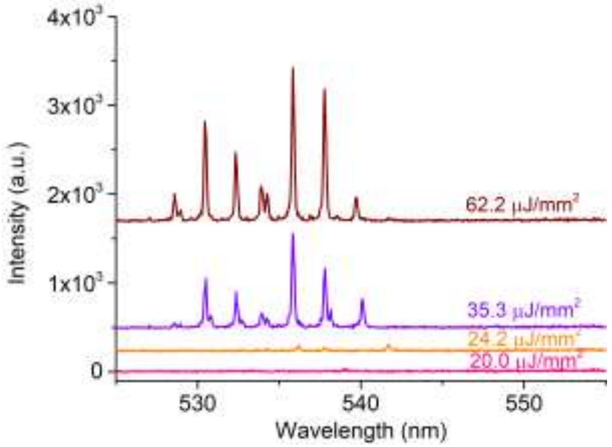

Supplementary Figure 2. Laser spectra with various pump energy density. Individual peaks appear with a pump energy density above the lasing threshold. The number of

longitudinal laser modes increases with an increasing pump energy density.

1.3 Influence of droplet size and cavity size on the laser mode pattern

As illustrated in Supplementary Fig. 1, the critical parameters of this configuration include the droplet size (*d*) and cavity size (*L*). The influence of these two parameters on the laser mode pattern is illustrated in Supplementary Figs. 3 to 4.

(1) Droplet size

Because of a lower intracavity gain in a small droplet, laser emission can hardly be achieved when the droplet size is below 10 μm. As illustrated in Supplementary Fig. 3, when the size of the droplets gets larger, a more complex transverse mode pattern can be observed. Even though more significant changes in transverse mode pattern are expected in a larger droplet size, the complex patterns make the mode identification very hard. The droplet size in the range 18-25 μm is preferred for laser mode measurement.

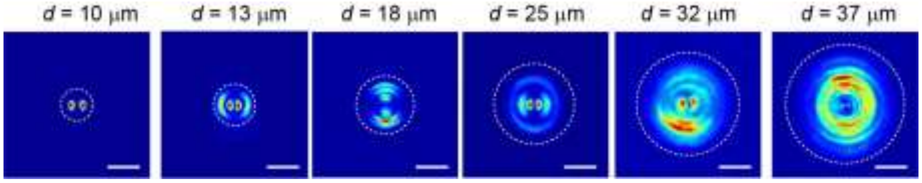

Supplementary Figure. 3. The laser mode pattern from LC droplets with different sizes. Scale bar: 10 μm. The doted circles denote the sizes of the droplets. The size of the cavity is kept at 45 μM.

(2) Cavity size

As illustrated in Supplementary Fig. 4, the cavity size shows no significant influence on the complexity of laser mode pattern. However, because of a higher lasing threshold induce by a larger intracavity loss with a larger cavity size, we do not suggest a cavity size larger than 50 μm.

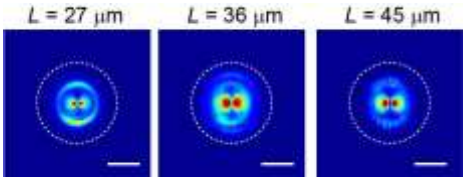

Supplementary Fig. 4. The laser mode pattern with different cavity sizes. The size of droplet is kept at 25 μm. Scale bar: 10 μm.

1.4 Ultrahigh sensitivity of laser modes

The polarized microscopy has been widely used as a gold-standard method for checking the conformation changes of LC. Here, we tried to compare the response of the laser mode to the conformation changes induced by biomolecules with the conventional polarized image. After BSA was induced, continues topological transformation of laser

mode can be observed (bottom, Fig. 2f). In contrary, the conventional polarized image remains stable (Supplementary Fig. 5) all the time.

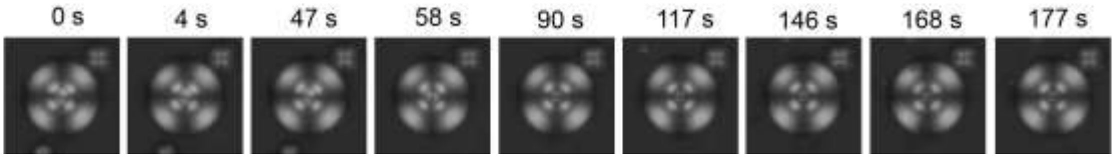

Supplementary Figure 5. Polarized pattern of LC droplet when 15 nM BSA was induced.

Then, we tried to increase the concentration of BSA to 150 nM and repeat the experiment. The results are given in Supplementary Fig. 6. Because the higher concentration tigers larger conformation changes in LC molecules, a more rapid transformation in laser mode was observed. However, the polarized image remains stable before 116.4 s. At 183.2s, significant changes in polarized image can be observed. In the meantime, the laser modes started to quench, which may come from an increasing intracavity loss in the radial to bipolar transition process. These results indicate that the laser mode possess much higher sensitivity than the polarized image, which is potentially used for molecular interaction investigation.

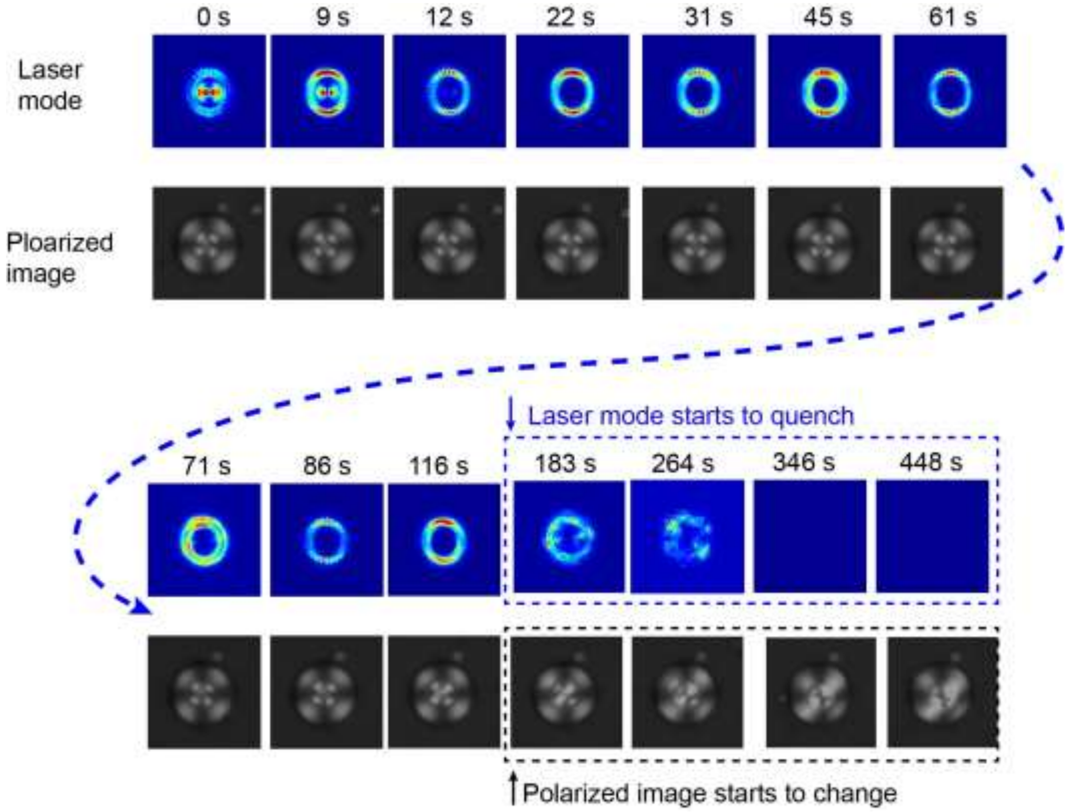

Supplementary Figure 6. Comparison between the laser mode evolution and the conventional polarized image. The BSA concentration is 150 nM.

2 INVESTIGATION OF AMYLOID PEPTIDE-LIPID MEMBRANE INTERACTIONS

2.1 Lipid coated LC droplets

A monolayer lipid self-assembled on the LC droplet was used to imitate the biological membrane. As illustrated in Supplementary Fig. 7a, once the 3 μM BSA was induced, the conformation of the LC droplet changes to bipolar immediately (within 1 s). However, when the uncoated LC droplet was used (the same as in Fig. 2), the polarized image of the LC droplet shows no significant conformation change (Supplementary Fig. 7b) even after 15 s. Note that the concentration of BSA is 20 times higher than used in Supplementary Fig. 6, which enables us to observe significant radial to bipolar changes in a shorter time. The result indicates the lipid coated LC droplets are more sensitive to molecular binding than the uncoated ones.

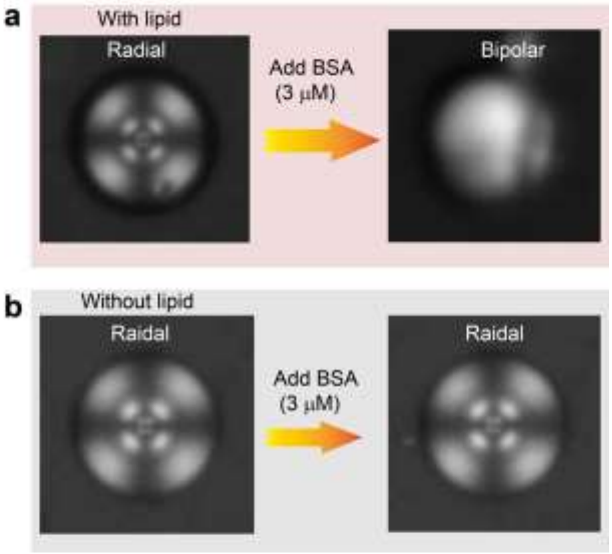

Supplementary Figure 7. **a**, The polarized image showing the conformation of lipid coated LC droplet changes from radial to bipolar when 3 μM BSA was added. **b**, The polarized image of uncoated LC droplet remaining the same after BSA was induced.

2.2 Recognition of the laser mode topologies

(1) Recognition of laser mode orders

The Laguerre-Gaussian modes are a series of solutions for paraxial wave equations in cylindrical coordinates, which can be written in terms of the Laguerre polynomials as[1]

$$u_{m,n}(r,\theta,z)=\sqrt{\frac{2m!}{(1+\delta_{0n})\pi(m+n)!}}\frac{1}{w(z)}\times\left(\frac{\sqrt{2}r}{w(z)}\right)^{n}L_{m}^{n}\left(\frac{2r^{2}}{w(z)^{2}}\right)e^{-ik\frac{r^{2}}{2q(z)}+in\theta} \quad (1)$$

where, *m*, *n* is the radial, azimuthal order of LG mode, respectively. $w(z)$,$q(z)$ is the size and the q-factor of the gaussian beam. L_m^n is Laguerre polynomial. δ_{0n} is defined as

$$\delta_{0n} = \begin{cases} 1 & n = 0 \\ 0 & n \neq 0 \end{cases} \tag{2}$$

Note that the guoy phase factor has been left out in Supplementary Eq. (1) for simplification. The electronic field of the observed laser mode is the linear combination of $\pm n$ terms $E_{m,n} = u_{m,n} + u_{m,-n}$. The laser mode pattern can be calculated using $I_{m,n} = \left|E_{m,n}\right|^2$. The LG mode with various orders was plotted in Supplementary Fig. 8, showing "dots" and "flowers" patterns with *m* and 2*n* node lines in radial and azimuthal direction. In particular, the observed "circles" in our experiment may be caused by the incoherent superposition of LC modes with different orders, thus making the azimuthal mode order unrecognizable.

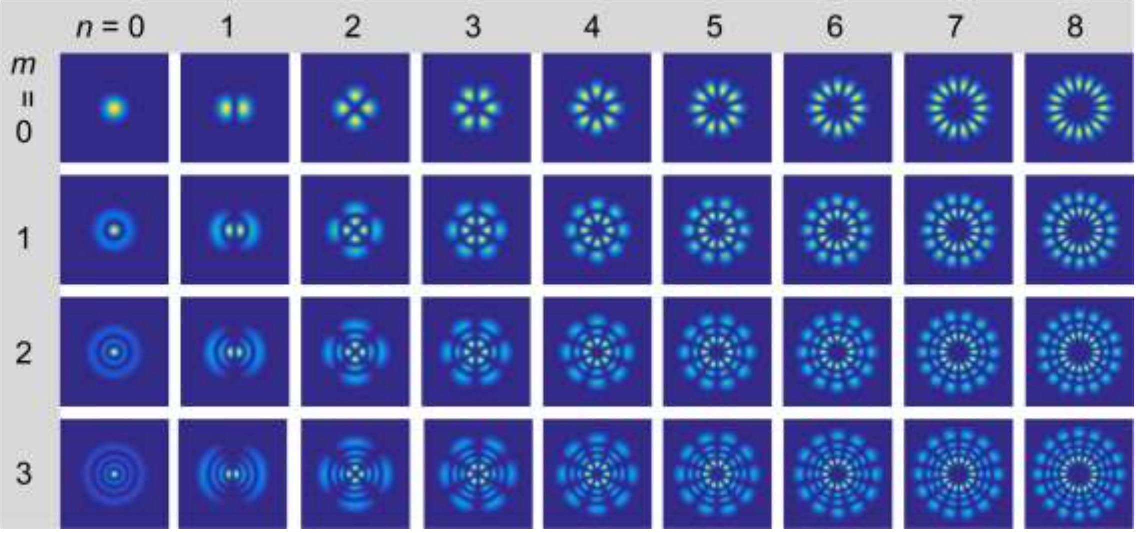

Supplementary Figure 8. LG mode patterns with various orders. The laser mode orders can be easily recognized by the node lines in the radial, azimuthal direction.

(2) Recognition of polarization

We have experimentally demonstrated a spatial dependent polarization vector in Fig. 2e. Here, we try to illustrate the feasibility of recognizing the polarization based on the laser mode pattern through a linear polarizer. In order to describe the polarization state of laser mode, we write the electronic filed of LG mode in a vector form

$$\vec{E}_{m,n} = \left(E^{r}_{m,n}, e^{-i\Delta\phi} E^{\theta}_{m,n}\right) \cdot \begin{pmatrix} \vec{e}_r \\ \vec{e}_\theta \end{pmatrix}, \tag{3}$$

where, $\vec{e}_r$ and $\vec{e}_\theta$ is the unit vector in radial and azimuthal direction. $E^{r}_{m,n}$ and $E^{\theta}_{m,n}$ is the amplitude in radial and azimuthal direction. $\Delta\phi$ is the phase difference between the radial and azimuthal direction. In Cartesian coordinates, the electronic filed can also be expressed as

$$\vec{E}_{m,n} = \left(A, B\right) \cdot \begin{pmatrix} \vec{e}_x \\ \vec{e}_y \end{pmatrix}, \tag{4}$$

where, $\vec{e}_x$ and $\vec{e}_y$ is the unit vector in the x and y-axis. *A* and *B* are the corresponding amplitude on the x and y-axis. According to Supplementary Fig. 9, we have

$$\begin{pmatrix} \vec{e}_x \\ \vec{e}_y \end{pmatrix} = \begin{pmatrix} \cos\theta & -\sin\theta \\ \sin\theta & \cos\theta \end{pmatrix} \cdot \begin{pmatrix} \vec{e}_r \\ \vec{e}_\theta \end{pmatrix} \tag{5}$$

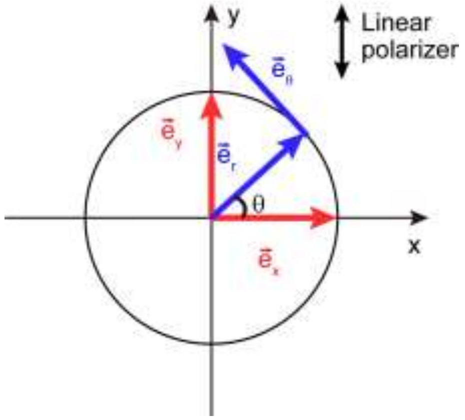

Supplementary Figure 9. Illustration of the correlation between $\vec{e}_x$, $\vec{e}_y$, $\vec{e}_r$, $\vec{e}_\theta$ and orientation of polarizer. By combing Supplementary Eqs. (3) to (5), the amplitude on x and y-axis can be written as

$$\left(A, B\right) = \left(E^{r}_{m,n}, e^{-i\Delta\phi} E^{\theta}_{m,n}\right) \cdot \begin{pmatrix} \cos\theta & \sin\theta \\ -\sin\theta & \cos\theta \end{pmatrix} \tag{6}$$

In our experiment, the orientation of the polarization is kept in parallel with the y-axis. The laser mode pattern after passing through a linear polarizer with a transmission axis in y direction can be written as

$$I^{y}_{m,n} = \left|B\right|^2 = \left|E^{r}_{m,n} \sin\theta + e^{-i\Delta\phi} E^{\theta}_{m,n} \cos\theta\right|^2 = \left|E^{r}_{m,n}\right|^2 \cdot \left|\sin\theta + \eta e^{-i\Delta\phi} \cos\theta\right|^2 \tag{7}$$

where $\eta = E^{\theta}_{m,n} / E^{r}_{m,n}$ denotes the ratio of the amplitude on the azimuthal and radial direction. By giving $E^{r}_{m,n} = E_{m,n}$, the laser mode patterns with various η and $\Delta\phi$ are plotted in Supplementary Fig. 10. After passing through a linear polarizer, two symmetry dark regions appear on the laser mode pattern, which is consistent with our experimental result.

The polarization of a specific laser mode can be easily recognized by the value of α, which is defined as the angle between the orientation of the maximum intensity and horizontal direction. As shown in Supplementary Fig. 10, $\pi/4 < |\pi - \alpha| \leq \pi/2$ corresponds to a radial polarization and $0 \leq |\pi - \alpha| < \pi/4$ corresponds to an azimuthal polarization. For simplification, we only recognize the polarization with the dominant amplitude. Specifically, when $|\pi - \alpha| = \pi/4$ or a uniform pattern as the central one in Supplementary Fig. 10 is observed, the laser mode pattern can be regarded as the combination of radial and azimuthal polarized modes with equal aptitude.

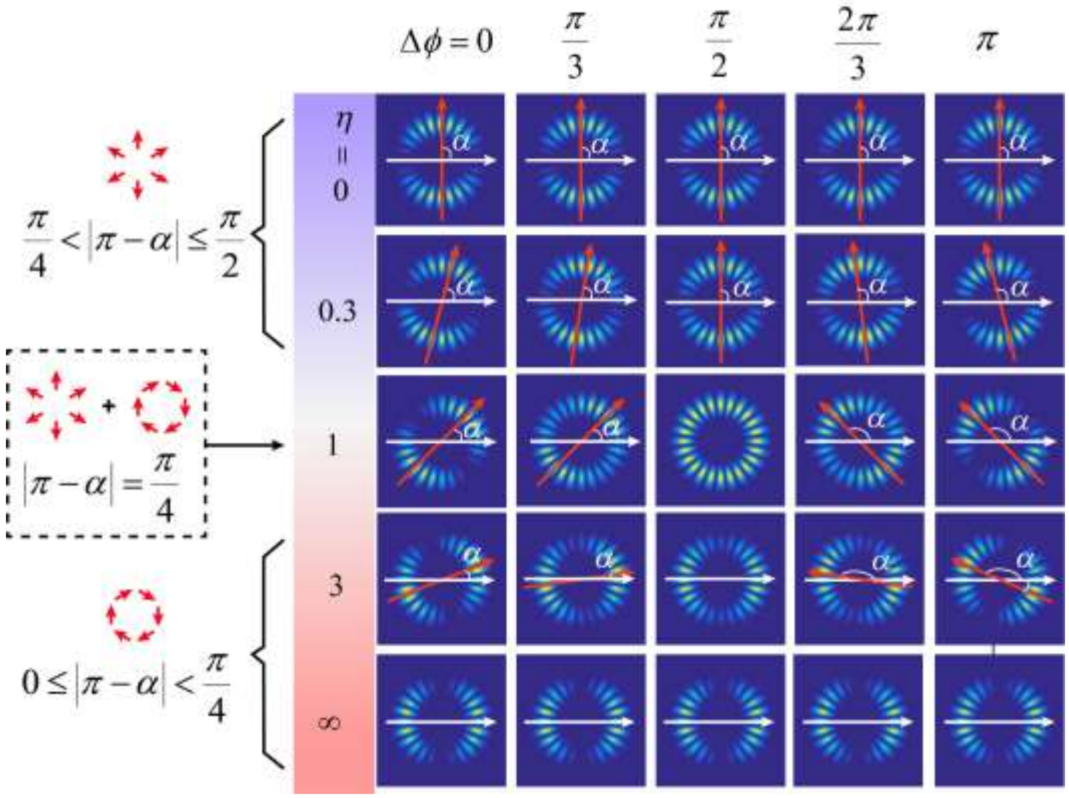

Supplementary Figure 10. Theoretical laser mode pattern after passing through a linear polarizer. α is defined as the angle between the orientation of the maximum intensity

and horizontal direction. $\eta = 0$ and $\eta = \infty$ corresponds to the situation when $E^{\theta}_{m,n} = 0$ and $E^{r}_{m,n} = 0$. The polarization state of the observed laser pattern can be easily determined by α.

2.3 Dynamic laser mode evolution with various Aβ peptide concentration

The laser mode evolution and the decomposition results with various Aβ peptide concentrations were recorded in Supplementary Figs. 11 to 13 and Fig. 3c. The corresponding topology parameters (*m*, *n*, *P*) were recorded in Supplementary Tables 1 to 4.

(1) 2.2 nM Aβ peptide

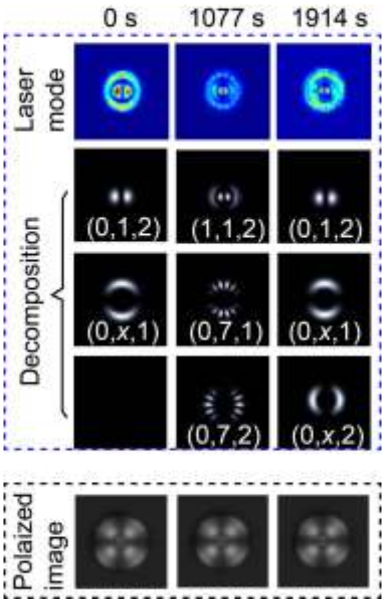

Supplementary Figure 11. Evolution of laser mode pattern with the decomposed

components (top) and the polarized images (bottom). The concentration of Aβ peptide is 2.2 nM.

Supplementary Table 1: Recording of laser mode evolution when Aβ peptide concentration is 2.2 nM.

LG modes	Topological parameters	0 s	1077 s		1914 s	
Dots (R)	*m*	0	1		0	
	n	1	1		1	
	P	2	2		2	
Flowers (G)	*m*		0	0		
	n		7	7		
	P		1	2		
Circles (B)	*m*	0			0	0
	n	*x*			*x*	*x*
	P	1			1	2

(2) 22 nM Aβ peptide

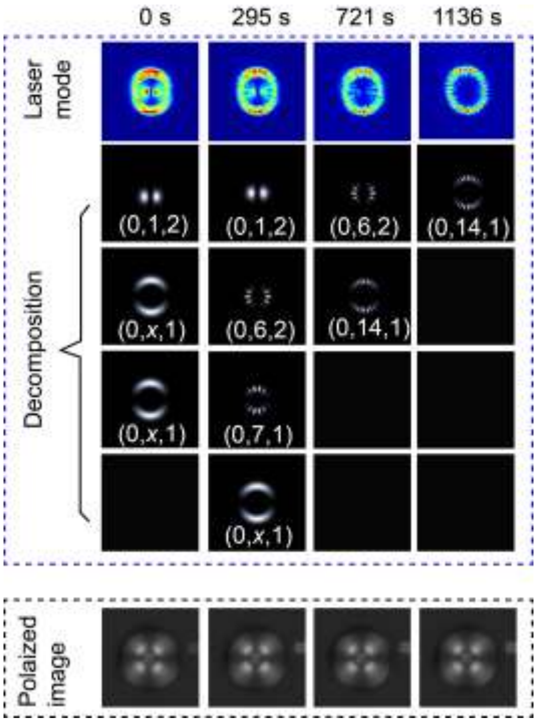

Supplementary Figure 12. Evolution of laser mode pattern with the decomposed components (top) and the polarized images (bottom). The concentration of Aβ peptide is 22 nM.

Supplementary Table 2: Recording of laser mode evolution when Aβ peptide concentration is 22 nM.

<table>
<tr><th>LG modes</th><th>Topological parameters</th><th colspan="2">0 s</th><th colspan="2">295 s</th><th colspan="2">721 s</th><th>1136 s</th></tr>
<tr><td rowspan="3">Dots (R)</td><td>m</td><td colspan="2">0</td><td colspan="2">0</td><td colspan="2"></td><td></td></tr>
<tr><td>n</td><td colspan="2">1</td><td colspan="2">1</td><td colspan="2"></td><td></td></tr>
<tr><td>P</td><td colspan="2">2</td><td colspan="2">2</td><td colspan="2"></td><td></td></tr>
<tr><td rowspan="3">Flowers (G)</td><td>m</td><td colspan="2"></td><td>0</td><td>0</td><td>0</td><td>0</td><td>0</td></tr>
<tr><td>n</td><td colspan="2"></td><td>6</td><td>7</td><td>6</td><td>14</td><td>14</td></tr>
<tr><td>P</td><td colspan="2"></td><td>2</td><td>1</td><td>2</td><td>1</td><td>1</td></tr>
<tr><td rowspan="3">Circles (B)</td><td>m</td><td>0</td><td>0</td><td colspan="2">0</td><td colspan="2"></td><td></td></tr>
<tr><td>n</td><td>x</td><td>x</td><td colspan="2">x</td><td colspan="2"></td><td></td></tr>
<tr><td>P</td><td>1</td><td>1</td><td colspan="2">1</td><td colspan="2"></td><td></td></tr>
</table>

(3) 220 nM Aβ peptide

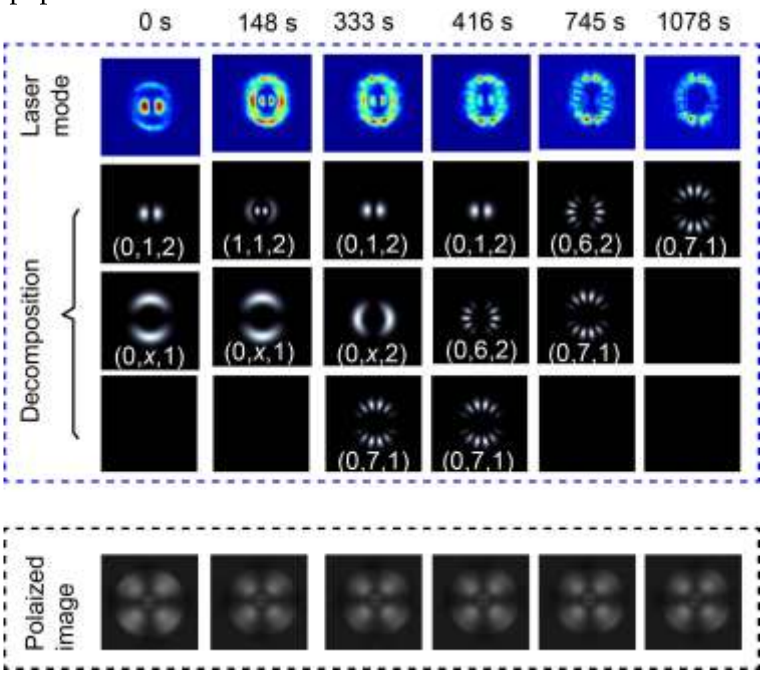

Supplementary Figure 13. Evolution of laser mode pattern with the decomposed components (top) and the polarized images (bottom). The concentration of Aβ peptide is 220 nM.

Supplementary Table 3: Recording of laser mode evolution when Aβ peptide

concentration is 220 nM.

LG modes	Topological parameters	0 s	148 s	333 s	416 s		745 s		1078 s
Dots (R)	*m*	0	1	0	0				
	n	1	1	1	1				
	P	2	2	2	2				
Flowers (G)	*m*			0	0	0	0	0	0
	n			7	6	7	6	7	7
	P			1	2	1	2	1	1
Circles (B)	*m*	0	0	0					
	n	*x*	*x*	*x*					
	P	1	1	2					

(4) 2.2 μM Aβ peptide

The laser mode evolution and the corresponding polarized images with 2.2 μM Aβ peptide is recorded in Fig. 3c.

Supplementary Table 4: Recording of laser mode evolution when Aβ peptide concentration is 2.2 μM.

LG modes	Topological parameters	0 s		57 s		238 s		299 s		327 s		426 s		506 s		600 s
Dots (R)	*m*			0		0						0		1		1
	n			1		1						1		1		1
	P			2		2						2		2		2
Flowers (G)	*m*			0	0			0	0	0	0	0	0	0	0	
	n			9	10			10	10	4	6	11	11	11	11	
	P			2	1			2	1	2	1	1	2	1	2	
Circles (B)	*m*	0	0			0	0			0						
	n	*x*	*x*			*x*	*x*			*x*						
	P	2	1			2	1			1						

3 LASER MODE MONITORING OF AMYLOID FIBRILLIZATION

Different Aβ assembly structures were obtained by incubating the Aβ peptide solution at 37°C for 0 h, 2h, 4 h, and 6 h (See Methods). Supplementary Figs. 14-16 show the laser mode evolution with Aβ after various incubation time. The topology parameters (*m*, *n*, *P*) were recorded in Supplementary Tables 5 to 7.

(1) 2h incubation

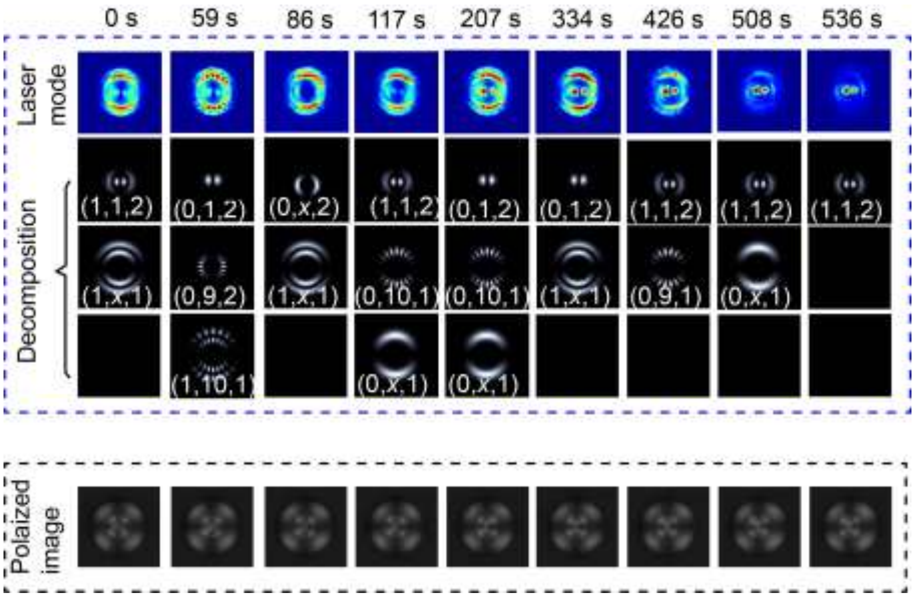

Supplementary Figure 14. Evolution of laser mode pattern and the decomposed components (top) and the polarized images (bottom). The Amyloid used in the experiment is incubated for 2h.

Supplementary Table 5: Recording of laser mode evolution with Aβ after 2 hours of incubation

<table>
<tr><th>LG modes</th><th>Topological parameters</th><th>0 s</th><th colspan="2">59 s</th><th colspan="2">86 s</th><th>117 s</th><th>207 s</th><th>334 s</th><th>426 s</th><th>508 s</th><th>536 s</th></tr>
<tr><td rowspan="3">Dots (R)</td><td>m</td><td>1</td><td colspan="2">0</td><td colspan="2"></td><td>1</td><td>0</td><td>0</td><td>1</td><td>1</td><td>1</td></tr>
<tr><td>n</td><td>1</td><td colspan="2">1</td><td colspan="2"></td><td>1</td><td>1</td><td>1</td><td>1</td><td>1</td><td>1</td></tr>
<tr><td>P</td><td>2</td><td colspan="2">2</td><td colspan="2"></td><td>2</td><td>2</td><td>2</td><td>2</td><td>2</td><td>2</td></tr>
<tr><td rowspan="3">Flowers (G)</td><td>m</td><td></td><td>0</td><td>1</td><td colspan="2"></td><td>0</td><td>0</td><td></td><td>0</td><td></td><td></td></tr>
<tr><td>n</td><td></td><td>9</td><td>10</td><td colspan="2"></td><td>10</td><td>10</td><td></td><td>9</td><td></td><td></td></tr>
<tr><td>P</td><td></td><td>2</td><td>1</td><td colspan="2"></td><td>1</td><td>1</td><td></td><td>1</td><td></td><td></td></tr>
<tr><td rowspan="3">Circles (B)</td><td>m</td><td>1</td><td colspan="2"></td><td>0</td><td>0</td><td>0</td><td>0</td><td>1</td><td></td><td>0</td><td></td></tr>
<tr><td>n</td><td>x</td><td colspan="2"></td><td>x</td><td>x</td><td>x</td><td>x</td><td>x</td><td></td><td>x</td><td></td></tr>
<tr><td>P</td><td>1</td><td colspan="2"></td><td>2</td><td>1</td><td>1</td><td>1</td><td>1</td><td></td><td>1</td><td></td></tr>
</table>

(2) 4 h incubation

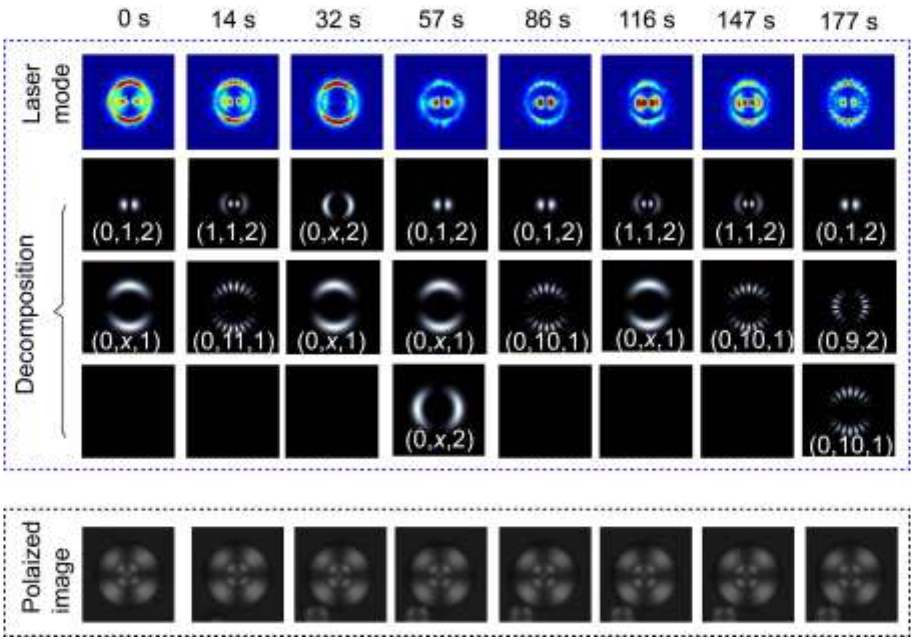

Supplementary Figure 15. Evolution of laser mode pattern and the decomposed components (top) and the polarized images (bottom). The Amyloid used in the experiment is incubated for 4h.

Supplementary Table 6: Recording of laser mode evolution with Aβ after 4 hours of incubation

<table>
<tr><th>LG modes</th><th>Topological parameters</th><th>0 s</th><th>14 s</th><th colspan="2">32 s</th><th colspan="2">57 s</th><th>86 s</th><th>116 s</th><th>147 s</th><th colspan="2">177 s</th></tr>
<tr><td rowspan="3">Dots (R)</td><td>m</td><td>0</td><td>1</td><td colspan="2"></td><td colspan="2">0</td><td>0</td><td>1</td><td>1</td><td colspan="2">0</td></tr>
<tr><td>n</td><td>1</td><td>1</td><td colspan="2"></td><td colspan="2">1</td><td>1</td><td>1</td><td>1</td><td colspan="2">1</td></tr>
<tr><td>P</td><td>2</td><td>2</td><td colspan="2"></td><td colspan="2">2</td><td>2</td><td>2</td><td>2</td><td colspan="2">2</td></tr>
<tr><td rowspan="3">Flowers (G)</td><td>m</td><td></td><td>0</td><td colspan="2"></td><td colspan="2"></td><td>0</td><td></td><td>0</td><td>0</td><td>0</td></tr>
<tr><td>n</td><td></td><td>11</td><td colspan="2"></td><td colspan="2"></td><td>10</td><td></td><td>10</td><td>9</td><td>10</td></tr>
<tr><td>P</td><td></td><td>1</td><td colspan="2"></td><td colspan="2"></td><td>1</td><td></td><td>1</td><td>2</td><td>1</td></tr>
<tr><td rowspan="3">Circles (B)</td><td>m</td><td>0</td><td></td><td>0</td><td>0</td><td>0</td><td>0</td><td></td><td>0</td><td></td><td colspan="2"></td></tr>
<tr><td>n</td><td>x</td><td></td><td>x</td><td>x</td><td>x</td><td>x</td><td></td><td>x</td><td></td><td colspan="2"></td></tr>
<tr><td>P</td><td>1</td><td></td><td>2</td><td>1</td><td>1</td><td>2</td><td></td><td>1</td><td></td><td colspan="2"></td></tr>
</table>

(3) 6 h incubation

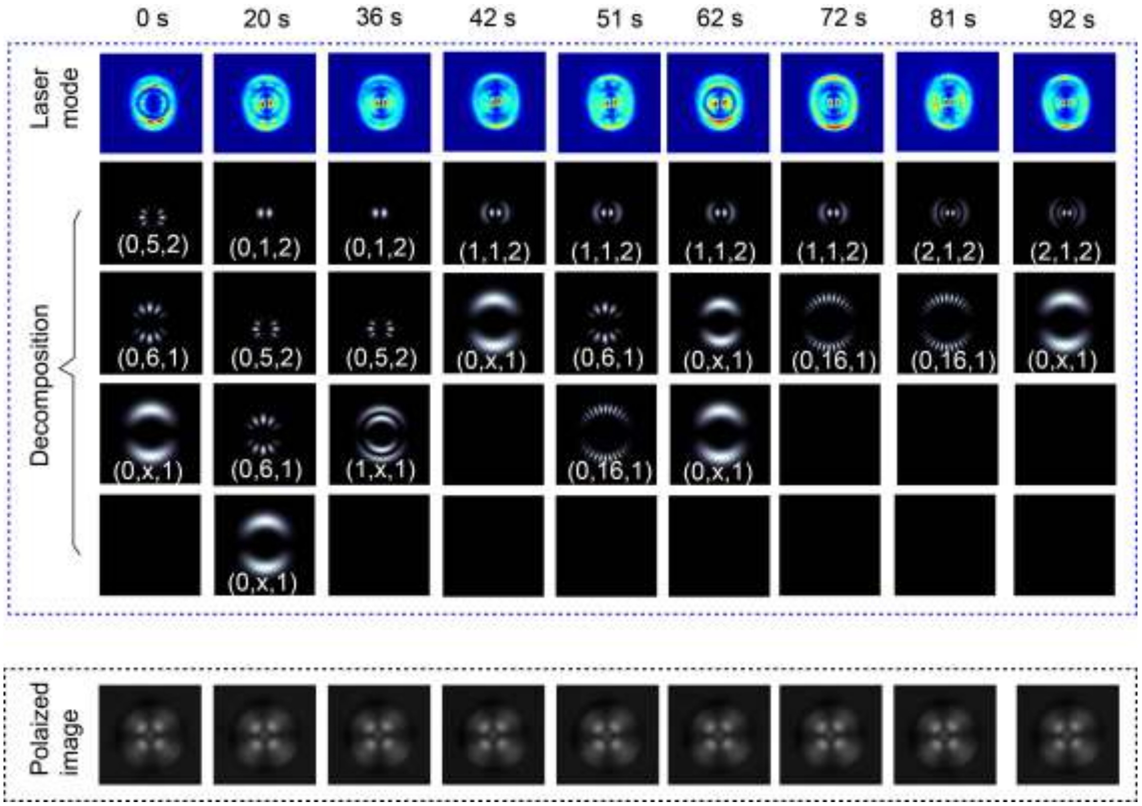

Supplementary Figure 16. Evolution of laser mode pattern and the decomposed components (top) and the polarized images (bottom). The Amyloid used in the experiment is incubated for 6h.

Supplementary Table 7: Recording of laser mode evolution with Aβ after 6 hours of incubation

<table>
<tr><th>LG modes</th><th>Topological parameters</th><th colspan="2">0 s</th><th colspan="2">20 s</th><th>36 s</th><th>42 s</th><th colspan="2">51 s</th><th colspan="2">62 s</th><th>72 s</th><th>81 s</th><th>92 s</th></tr>
<tr><td rowspan="3">Dots (R)</td><td>m</td><td colspan="2"></td><td colspan="2">0</td><td>0</td><td>1</td><td colspan="2">1</td><td colspan="2">1</td><td>1</td><td>2</td><td>2</td></tr>
<tr><td>n</td><td colspan="2"></td><td colspan="2">1</td><td>1</td><td>1</td><td colspan="2">1</td><td colspan="2">1</td><td>1</td><td>1</td><td>1</td></tr>
<tr><td>P</td><td colspan="2"></td><td colspan="2">2</td><td>2</td><td>2</td><td colspan="2">2</td><td colspan="2">2</td><td>2</td><td>2</td><td>2</td></tr>
<tr><td rowspan="3">Flowers (G)</td><td>m</td><td>0</td><td>0</td><td>0</td><td>0</td><td>0</td><td></td><td>0</td><td>0</td><td colspan="2"></td><td>0</td><td>0</td><td></td></tr>
<tr><td>n</td><td>5</td><td>6</td><td>5</td><td>6</td><td>5</td><td></td><td>6</td><td>16</td><td colspan="2"></td><td>16</td><td>16</td><td></td></tr>
<tr><td>P</td><td>2</td><td>1</td><td>2</td><td>1</td><td>2</td><td></td><td>1</td><td>1</td><td colspan="2"></td><td>1</td><td>1</td><td></td></tr>
<tr><td rowspan="3">Circles (B)</td><td>m</td><td colspan="2">0</td><td colspan="2">0</td><td>1</td><td>0</td><td colspan="2"></td><td>0</td><td>0</td><td></td><td></td><td>0</td></tr>
<tr><td>n</td><td colspan="2">x</td><td colspan="2">x</td><td>x</td><td>x</td><td colspan="2"></td><td>x</td><td>x</td><td></td><td></td><td>x</td></tr>
<tr><td>P</td><td colspan="2">1</td><td colspan="2">1</td><td>1</td><td>1</td><td colspan="2"></td><td>1</td><td>1</td><td></td><td></td><td>1</td></tr>
</table>
